# Synthesizing CuO/CeO_2_/ZnO Ternary Nano-Photocatalyst with Highly Effective Utilization of Photo-Excited Carriers under Sunlight

**DOI:** 10.3390/nano10101946

**Published:** 2020-09-29

**Authors:** Kaiyi Luo, Jing Li, Wenyu Hu, Han Li, Qiuping Zhang, Huan Yuan, Fei Yu, Ming Xu, Shuyan Xu

**Affiliations:** 1 College of Electrical & Information Engineering & Key Lab of Information Materials of Sichuan Province, Southwest University for Nationalities, Chengdu 610041, China; kaiyi_luo@163.com (K.L.); hedima123@163.com (J.L.); h17708055315@163.com (W.H.); lllhan0922@163.com (H.L.); zhqpdq@163.com (Q.Z.); yuanh@uestc.edu.cn (H.Y.); yufei@swun.edu.cn (F.Y.); 2 Plasma Sources and Application Center, Nanyang Technological University, Singapore 37616, Singapore; shuyan.xu@nie.edu.sg

**Keywords:** CuO/CeO_2_/ZnO, heterostructured photocatalyst, effective charge separation

## Abstract

The construction of heterostructured photocatalyst with an appropriate energy band structure will help realize highly efficient photo-excited charge separation. In this study, ternary CuO/CeO_2_/ZnO nano-particle (NP) composites were synthesized by a facile two-step sol-gel method, which exhibit significantly enhanced photocatalytic degradation performance for various organic pollutants under UV and visible light excitation. The photo-responses to both UV and visible light, as well as the visible light absorption and utilization rates of ZnO are found to be synergistically intensified by CeO_2_ and CuO co-coupling. The first-order kinetic constants (K) of 3%CuO/CeO_2_/ZnO for methylene blue (MB) degradation are ~3.9, ~4.1 and ~4.8 times higher than ZnO under UV light, visible light and simulated sunlight illumination, respectively. The roles of CuO and CeO_2_ in optical properties and photo-degradation under UV and visible light were explored. Besides, the photogenic holes (h^+^) of ZnO, CeO_2_, and the produced hydroxyl radicals (·OH) are proved to be the main active species under UV light. Dissimilarly, under visible light, the superoxide radicals (·O_2_^−^) formed by the reactions between oxygen molecules and the photo-generated electrons (e^−^) of CuO moving towards the catalysts surface are also found to be important for promoting dye decomposition. The improved photo-responses, the well-matched band structure that facilitates charge transfer processes, and the highly efficient utilization of the photo-excited carriers of the ternary nano-heterostructure are suggested to be the key factors for the remarkable enhancement of photocatalytic performance of ZnO nano-photocatalyst. This work offers a low-cost strategy to acquire highly active UV and visible light-driven photocatalyst.

## 1. Introduction

Demands of the tangible current severe worldwide situation, from cleaning, energy harvesting to environment remediation techniques, are expecting the development and adoption of highly efficient photocatalytic system [1]. The exploiting of high-quality and cost-effective photocatalytic materials is the most pivotal part of this system. Classical wide band-gap metal oxide semiconductors (MOSs), such as ZnO (Eg = 3.37 eV), TiO_2_ (Eg = 3.20 eV), SnO_2_ (Eg = 3.80 eV), etc., cannot meet the requirements of a superior catalytic platform because of the limited sunlight utilization and low photo-generated charge carrier separation efficiency [1,2,3].

Currently, numerous approaches have been explored to extend the solar absorption range of those MOSs, as well as improve the lifetime of photo-excited charge carriers. The common treatments to solve these problems are correlated with “defect engineering” [4]. The band structure of as-treated semiconductor can be maneuvered by the defects states to extend light response range, meanwhile surface defects serve as highly active sites for photocatalytic reactions [4]. Methods like surface hydrogenation [1], metal reduction [5], are representative defect engineering strategies. In spite of the high activity of as-treated photocatalysts, these methods rely on cumbersome and expensive synthetic craft.

Heterostructure engineering is considered as another effective way to acquire photocatalytic materials with fine optical and electrical properties [6,7,8]. In this type of composite structure with enhanced photocatalytic performance, each component possesses theoretically ideal energy band position to approve charge transfer between the coterminous semiconductors, and promote photocatalytic oxidation and reduction reactions by accelerating the formation of strong oxidizing radicals (·OH, O_2_^−^, etc.). For instance, by decorating precious metals such as Au, Ag on the MOSs (TiO_2_, ZnO, etc.) surface, the light absorption capacity, as well as the photo-response of semiconductor under UV and visible light excitation can be significantly enhanced because of the existence of schottky barrier and surface plasmon resonance (SPR) intrinsic to the metal [2,9,10,11]. However, metal incorporation cannot realize industrialization on account of high production costs. For this reason, construction of heterostructure composed by semiconductors is more exercisable in comparison. Cerium dioxide (CeO_2_, E_g_ = 2.58 eV), an excellent rare-earth oxide semiconductor, has been shown to be a particularly effective catalyst [12,13]. Considering the conduction band (CB) and valence band (VB) potentials of CeO_2_, it is a suitable semiconductor for coupling with ZnO to form heterogeneous structure with high charge transfer efficiency. Besides, copper oxide (CuO, Eg = 1.40 eV) coupling was found to be an effective manner to extend the spectral absorption and response of ZnO [14]. On this basis, by decorating precious metals (such as Ag, Au, etc.) on CuO/ZnO, the ternary composite photocatalysts with more efficient photocatalytic performance emerged afterwards [15,16]. In this work, we explore whether CuO, CeO_2_ co-coupled ZnO, can analogously exhibit improved photocatalytic efficiency as a ternary hetero-structured photocatalyst system. Notably, despite some work reported on CuO/ZnO and CeO_2_/ZnO photocatalysts [12,14], however, the inherent relations between the photocatalytic properties of CuO/CeO_2_/ZnO and each component remain unclear [17,18].

Frequently used manners to fabricate semiconducting heterosturcture photocatalyst include impregnation, co-precipitation, hydrothermal method, vapor deposition for 2-demensional (2D) materials fabrication [19,20,21,22], to name but a few. In our previous work [15], sol-gel technique was evidenced to be a facile and low-cost method to fabricate highly efficient composite photocatalyst. Herein, we established a low-cost and highly active CuO/CeO_2_/ZnO heterostructured photocatalytic platform for exploring the internal connections among the components. Specifically, the designed ternary structure with significantly enhanced photo-excited charge separation efficiency was synthesized by a simple two-step sol-gel method. By investigating the efficiency of ZnO, CeO_2_/ZnO and CuO/CeO_2_/ZnO for the photo-degradation of various organic contaminants (methylene blue (MB), methyl orange (MO) and rhodamine b (RhB)) under UV light, visible light and simulated sunlight illumination, the photocatalytic performance of ZnO was proved to be synergistically improved by CeO_2_ and CuO introduction. The effects of CeO_2_ and CuO co-coupling on light absorption, photo-responses and the photo-generated e^−^/h^+^ pairs recombination processes were explored. In addition, the efficient utilization of the photo-excited carriers in photocatalytic reactions was revealed.

## 2. Materials and Methods

### 2.1. Synthesis of CuO/CeO_2_/ZnO Nano-Composites

All chemical reagents used in the synthesis were of analytical grade and can be directly used without further purification. The CuO/CeO_2_/ZnO nano-composites were synthesized by a facile two-step sol-gel method. Zn, Ce, Cu elements were respectively sourced from zinc acetate dihydrate (Zn(CH_3_COO)_2_·2H_2_O, Chengdu Kelon Chemical Reagent Co., LTD., Chengdu, China, ≥99.0%), cerium nitrate hexahydrate (Ce(NO_3_)_3_·6H_2_O, Chengdu Kelon Chemical Reagent Co., LTD., ≥99.0%) and copper acetate monohydrate (Cu(CH_3_COO)_2_·H_2_O, Chengdu Kelon Chemical Reagent Co., LTD., ≥99.0%) to prepare ZnO, CeO_2_/ZnO and CuO/CeO_2_/ZnO nano-particles (NPs). Typically, precursors (Zn = 0.02 mol, Ce = 3% × 0.02 = 0.0006 mol) and 2 mL precipitant diethanol amine (C_4_H_11_NO_2_, Chengdu Kelon Chemical Reagent Co., LTD., ≥85.0%) were dispersed into 60 mL ethyl alcohol (C_2_H_6_O, Tianjin Zhiyuan Chemical Reagent Co., LTD., Tianjin, China, ≥99.7%), followed by mixing and stirring in a water bath at 60 °C for 2 h and then a stable clear sol system formed. After standing for 48 h, the resultant gel was dried at 80 °C and then calcinated at 450 °C for 10 h, CeO_2_/ZnO NPs were obtained. Then, *x*% × 0.02 (*x* = 1, 3) mol of Cu precursor was added into the resultant products and the above processes were repeated. The samples were denoted as ZnO, 3%CeO_2_/ZnO, 1%CuO/CeO_2_/ZnO and 3%CuO/CeO_2_/ZnO according to the constituent and CuO content. The specific reagent masses used in fabrication processes are presented in Table 1.

### 2.2. Characterization

The crystalline micro-structure studies were carried out in a DX-2000 powder X-ray diffraction (XRD) instrument, the incident X-ray energy was measured as 3 kV with incident angle ranging from 20° to 80°. A JEOL JSM-7500F c-FEG scanning electron microscope (SEM) operated at 15 keV was used to observe the surface morphology of the nanocrystals. Transmission Electron Microscope (TEM), scanning TEM (STEM), energy dispersive spectroscopy (EDS) and high-resolution TEM (HRTEM) were performed on a FEI (Thermo Fisher) Tecnai G2 F20 microscope operated at 200 kV acceleration voltage. The light absorption capacities of the materials were characterized by a UV-vis spectroscopy (UV-vis, UV-2550) with an integral sphere. The surface photovoltage (SPV) surface photocurrent (SPC) properties were detected by a home-built instrument (Jilin University) consisting of a source of monochromatic light, a lock in amplifer (SR830-DSP) with a light chopper (SR540) and a photovoltaic cell at room temperature. The steady-emission properties were evaluated by a PekinElmer FL-8500 photoluminescence spectroscopy at the excitation wavelength of 325 nm.

### 2.3. Photocatalytic Experiments

The photocatalytic degradation properties of CuO/CeO_2_/ZnO ternary composites were explored by considering the degradation of methylene blue (MB), methyl orange (MO) and rhodamine B (RhB) under simulated sunlight, UV and visible light illumination. Initial absorbance (A_0_) scanning of the dye (4 mg·L^−1^) solution was performed at their characteristic wavelength of 664, 464 and 554 nm, respectively. Typically, 50 mg of sample was dispersed in 100 mL MB/MO/RhB solution. After 10 min of ultrasonic oscillation, the sample-dye suspension was left in a dark environment and stood for 20 min to satisfy the adsorption–desorption equilibrium. The solution with photocatalyst was then irradiated by using a high-pressure mercury lamp and xenon lamp, the irradiation distance was 20 cm, and the irradiation intensity was 0.17 and 0.03 W·cm^−2^ for a xenon lamp and high-pressure mercury lamp, respectively. A total of 5 mL of the residual contamination solution was taken out at regular intervals and was then centrifuged at a speed of 6000 r·min^−1^ using a centrifugal machine, the supernatant was collected and the absorbance (A_t_) was measured by spectrophotometer.

The real-time degradation rate (Y_t_) and kinetic degradation constants (K_t_) are calculated by the following relations:

Y_t_ = (C_0_ − C_t_)/C_0_ × 100% = (A_0_ − A_t_)/A_0_ × 100%, (1)

K_t_ = ln (C_0_/C_t_), (2)

where C_0_ represents the initial concentration, and C_t_ denotes the measured concentration.

The in situ MB emission characterizations were performed using PerkinElmer 8500-F photoluminescence spectroscopy. A 150 W Xenon lamp equipped with a dispersive optical grating and a 150 mm optical slit was used to provide the excitation system, which allows for the selection of a certain monochromatic illumination wavelength from 200 to 900 nm spectrum ranges. A 150 mm entrance slit with an optical grating coupled system provides a maximum 200~900 nm spectral range with a wavelength resolution (FWHM of excitation peak) of about 10 nm. The samples (50 mg)/MB suspension (4 mg/mL, 100mL) was prepared by ultrasonic concussion. The sample/solution suspension was kept in a dark for 20 min. A cuvette was used as a container for sample/MB solution mixture. The acquisition duration was set as 1.5 min/per spectrum to obtain in situ spectra series. Cycling photocatalytic experiments are conducted to investigate the stability of the nano-photocatalyst. After each run of dye degradation, the sample was recycled by a series of procedures, including filtration, washing and drying, and applied for the next run.

A total of 0.2 mmoL Benzoquinone (BQ, Chengdu Huaxia Chemical Reagent Co., LTD., Chengdu, China, ≥99.0%), 0.5 mmoL edta-disodium (EDTA-2Na, Tianjin Fuchen Chemical Reagent Co., LTD., Tianjin, China, ≥99.0%) and 0.5 mmoL isopropyl alcohol (IPA, Zhiyuan Chemical Reagent Co., LTD., ≥99.7%) were added in the experiment of degradation of MB as trapping agents to trap superoxide radical (·O_2_^−^), hole (h^+^) and hydroxyl radical (OH), respectively.

## 3. Results and Discussion

### 3.1. Structural and Morphological Study

**Figure 1:**
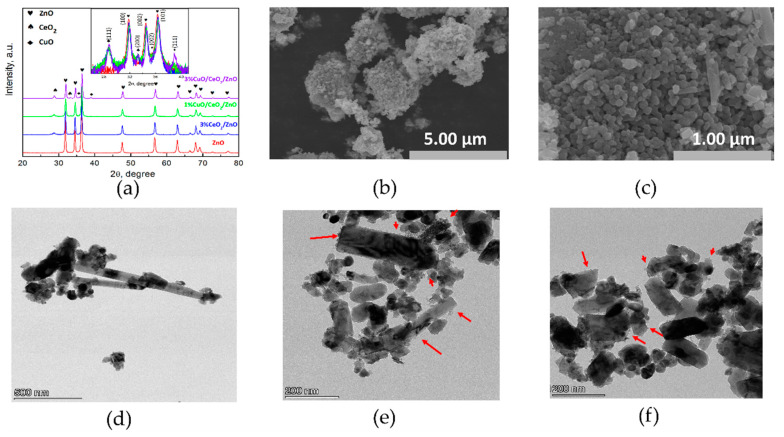
(**a**) X-ray Diffraction (XRD) patterns of the as-synthesized samples. (**b**,**c**) Scanning Electron Microscope (SEM) images of sample 3%CuO/CeO_2_/ZnO; (**d**–**f**) Transmission Electron Microscope (TEM) images of sample 3%CuO/CeO_2_/ZnO.

The crystalline structure of pure ZnO, CeO_2_/ZnO and CuO/CeO_2_/ZnO composites were examined by X-ray diffraction (XRD), as shown in Figure 1a. The strong diffraction peaks of all the synthesized samples can be indexed as the hexagonal wurtzite phase of ZnO (JCPDS file no. 36-1451). The diffraction peaks at 2θ of 28.62° and 33.17°, respectively, correspond to (111) and (200) planes of fluorite cubic CeO_2_ (JCPDS files no. 34-0394). The peaks located at 35.72° and 38.79° are assigned to (002) and (111) crystal planes of tenorite CuO (JCPDS files no.48-1548). Compared to pure ZnO, a slight decrease in the diffraction angles is observed after the introduction of CeO_2_ and CuO. The lattice constants (a and c), and cell volume of hexagonal wurtzite structure were calculated by Equations (3) and (4):

1/d^2^ = 3/4 [(h^2^ + hk + k^2^)/a^2^] + (l^2^/c^2^) (3)

V = 0.866 × a^2^ × c (4)

The crystal size of ZnO was determined by Scherrer’s equation, as shown in Equation (5):

D = kλ/βcosθ(5)

where k, λ, and β are shape factor (0.89), the wavelength of X-ray and full width at half maximum (FWHM) of correlation peaks, respectively.

The micro-structure parameters of the as-synthesized samples are presented in Table 2. With the coupling of CeO_2_ and CuO, the lattice constants (a, c) of ZnO decrease and jointly, the cell volume shrinks. The reduction in the peak intensity indicates the declined crystallinity of ZnO in the composite samples. The average crystal size of ZnO were calculated to range from 28.6 to 36.4 nm, suggesting that the grain growth of ZnO might be inhibited by the introduction of CeO_2_, CuO.

**Figure 2:**
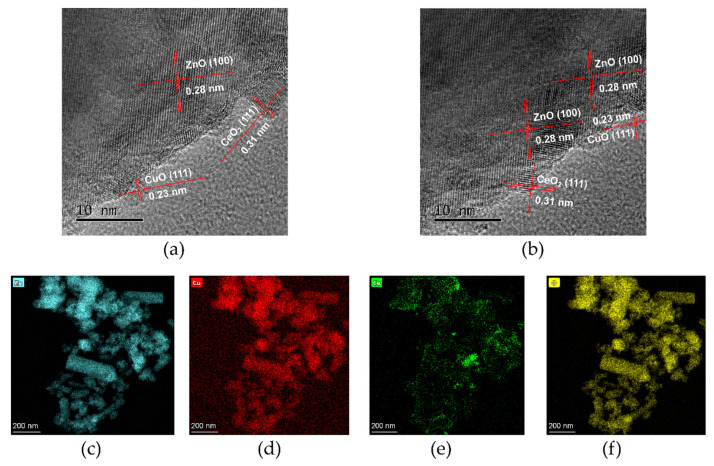
(**a**,**b**) High-Resolution Transmission Electron Microscope (HR-TEM) images of sample 3%CuO/CeO_2_/ZnO; (**c**–**f**) element mapping results of sample 3%CuO/CeO_2_/ZnO.

Scanning electron microscope (SEM) images (Figure 1b,c) display the homogeneous distribution and the spherical and rod-shaped hybrid morphology of the as-synthesized composites (3%CuO/CeO_2_/ZnO). The rod-like morphology of ZnO is attributed to the growth of the crystal along the c-axis [23,24], which may lead to a highly efficient photocatalytic reaction due to the large surface area of such structure as compared to nanoparticle [25]. As shown in Figure 1d–f, the transmission electron microscopy (TEM) images evidently show that the nanorod structure existed in the sample, and quite a few small-size particles were observed on the surface of ZnO. Furthermore, the high-resolution TEM (HR-TEM) images shown in Figure 2a,b evidence the successful construction of CuO/CeO_2_/ZnO ternary nano-heterostructure, and the interplanar spacings of 0.28, 0.23, and 0.31 nm, respectively, correspond to the (100), (111) and (111) crystal planes of ZnO, CuO and CeO_2_ [13,21]. The microstructure and compositional distribution of the nanocrystals were further investigated by scanning TEM (STEM) and energy dispersive spectroscopy (EDS). The element mapping images (Figure 2c–f) approve the complete overlapping of three semiconductor components. The full coverage of Zn, Cu, Ce, O indicates that the four elements are uniformly distributed in the sample.

### 3.2. Optical Study

**Figure 3:**
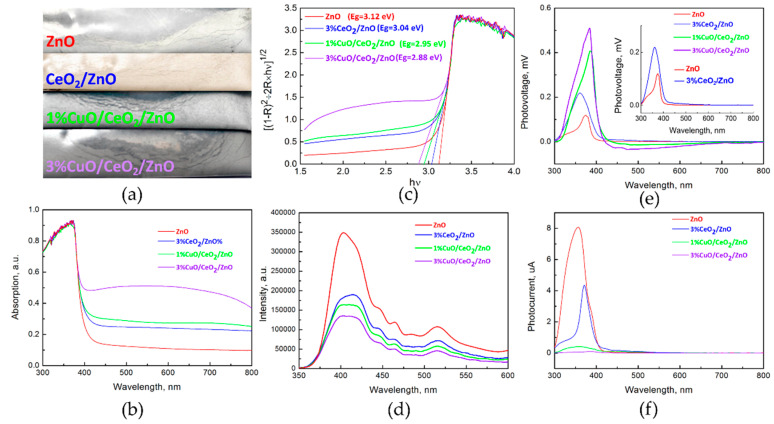
(**a**) Appearances of the as-synthesized samples. (**b**) UV-vis absorption spectra of the as-synthesized samples. (**c**) Band-gap widths calculated from Kubelka-Munk function. (**d**) Photoluminescence (PL) spectra of the as-synthesized samples. (**e**) Surface photovoltage (SPV) and (**f**) surface photocurrent (SPC) spectra of the samples.

Figure 3a exhibits the color evolution of of the sample after introducing CeO_2_ and CuO. The samples gradually changed from white (pure ZnO) to dark grey. UV-vis spectra (Figure 3b) reveal that the spectral absorption capacities of ZnO are significantly boosted by CeO_2_ and CuO co-coupling. A ~2-fold enhancement of visible light absorption of CeO_2_/ZnO is observed when 3 mol% CuO was introduced. According to Kubelka-Munk function, the band-gap widths (Figure 3c) of the samples were calculated to be 3.12, 3.04, 2.95, and 2.88 eV for ZnO, 3%CeO_2_/ZnO, 1%CuO/CeO_2_/ZnO and 3%CuO/CeO_2_/ZnO, respectively. The reduced band-gap width caused by CuO and CeO_2_ introduction can make the electrons be excited by lower incident energy, thereby improving the light utilization efficiency. Photoluminescence (PL) refers to the process of using Xe lamp as an excitation source to excite electrons in a material and the light emission can be achieved by the recombination of photo-generated charge carriers. The PL spectra are presented in Figure 3d. One can see that the PL intensity of ZnO was explicitly reduced step by step, implying the recombination of photo-excited e^−^/h^+^ pairs of ZnO was suppressed by coupling with CeO_2_ and CuO. The UV emission can be attributed to the recombination of free excitons formed by the band-to-band transition of ZnO. The visible light emission is attributed to the radiative transitions among the impurity levels (such as Zn_i_, V_Zn_, V_o_, etc.). The common luminescence centers of ZnO nanocrystal were discussed in detail in our previous work [10,15]. Surface photo-voltage (SPV) technique was adopted to further analyze the photo-generated charge carrier transfer and recombination. As displayed in Figure 3e, the intensified SPV signals evidence the reduced recombination rate of photo-excited e^−^/h^+^ pairs. The inset in Figure 3e clearly shows the dissimilitude between ZnO and 3%CeO_2_/ZnO samples. A slight improvement of visible light response (~400 nm to ~600 nm) is found when CeO_2_ was introduced. As CeO_2_ is a n-type semiconductor with a ~2.58 eV band-gap width, the build-in electrical field in the surface space charge region orients from bulk towards surface and the photo-generated h^+^ moves to the catalyst surface, so a positive signal is observed in the visible region [12,26,27,28]. Interestingly, a negative SPV signal rises in the visible range (~400 nm to ~800 nm) when 1 mol% CuO was added, and further enhances as increasing CuO content. The negative photo-voltage means that the photo-generated e^−^ of CuO (*p*-type) transfers from the bulk to the surface of the samples under visible light illumination [27]. Figure 3f shows the surface photo-current (SPC) spectra of the as-synthesized samples. The decreased photo-current is attributed to the decreased amount of free carriers in the heterostructures [9]. Hence, the synergistic effects of CuO and CeO_2_ on optical and electrical properties of ZnO are summarized as follows: (1) Reducing the band-gap width of ZnO and enhancing the visible light absorption (2) Improving both UV and visible light responses. (3) Suppressing the recombination of photo-generated electron-hole pairs. However, CuO supplies much higher visible light absorption rate than CeO_2_, and leads to an exactly converse type of photo-response in the visible range.

### 3.3. Photocatalytic Activity Study

**Figure 4:**
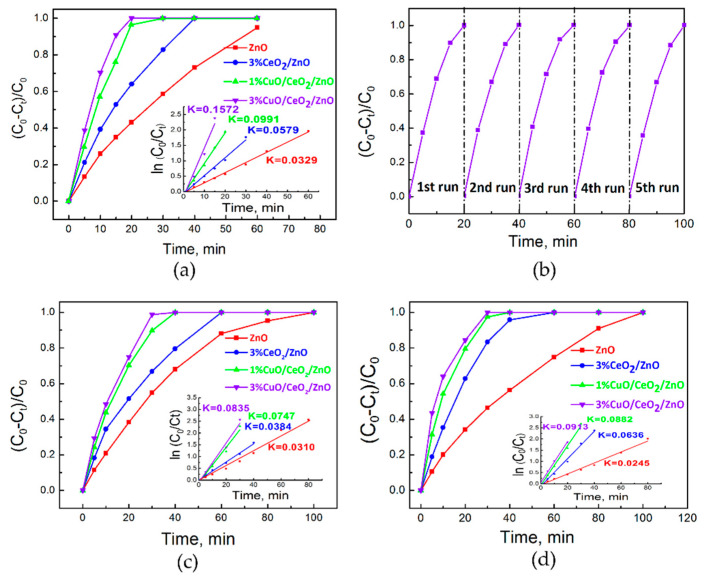
Photocatalytic degradation curves and the first-order kinetic constants (ln (C_0_/C_t_)) of the as-synthesized samples for methylene blue (MB), methyl orange (MO) and rhodamine b (RhB) decomposition. (**a**) Photocatalytic degradation of MB investigated under simulated sunlight irradiation. (**b**) Cycling experiments of MB degradation under simulated sunlight with sample 3% CuO/CeO_2_/ZnO. (**c**,**d**) Photocatalytic degradation of MO, RhB, respectively, investigated under simulated sunlight irradiation.

Photocatalytic performance was evaluated by considering the degradation of various organic dyes (MB, MO and RhB). As displayed in Figure 4a, the degradation rates of MB in 15 min under simulated sunlight are 91%, 76%, 53% and 35% for 3%CuO/CeO_2_/ZnO, 1%CuO/CeO_2_/ZnO, 3%CeO_2_/ZnO, ZnO, respectively. The kinetic degradation constant of 3%CuO/CeO_2_/ZnO (K = 0.1572 min^−^^1^) is ~4.8 times higher than that of ZnO. Cycling experiments for sample 3%CuO/CeO_2_/ZnO (Figure 4b) were conducted and the results indicate the performance of the photocatalyst is stable. Moreover, we measured the degradation of MO and RhB under simulated sunlight. As expected, the order of photocatalytic activities is 3%CuO/CeO_2_/ZnO > 1%CuO/CeO_2_/ZnO > 3%CeO_2_/ZnO > ZnO, as shown in Figure 4c,d. Moreover, sample 3%CuO/CeO_2_/ZnO exhibits the most efficient performance for MO and RhB degradation.

**Figure 5:**
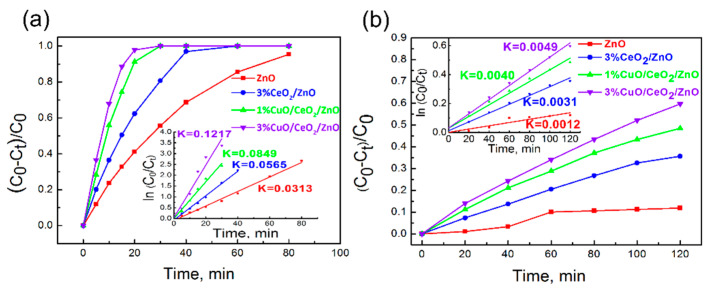
(**a**) Photocatalytic degradation curves of MB and the *K*-values investigated under UV light irradiation. (**b**) Photocatalytic degradation curves of MB and the *K*-values investigated under visible light (λ > 400 nm). Irradiation time: 120 min.

Photocatalytic experiments of MB degradation were also conducted under UV and visible light irradiation (λ > 400 nm). Figure 5a,b show that the photocatalytic activity of CuO/CeO_2_/ZnO ternary heterostructure is significantly improved compared to ZnO and binary CeO_2_/ZnO nano-composites under both UV and visible light. The photocatalytic efficiency of 3%CuO/CeO_2_/ZnO under UV light is ~3.9 and ~2.2 times higher than ZnO and 3%CeO_2_/ZnO, and is ~4.1 and ~1.6 times higher than ZnO and 3%CeO_2_/ZnO under visible light, respectively.

**Figure 6:**
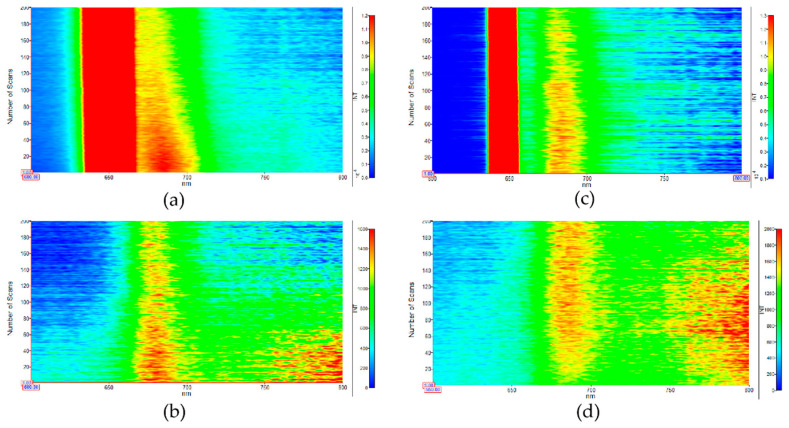
(**a**,**b**) In situ MB emission images for 3%CuO/CeO_2_/ZnO under the excitation wavelengths of 325 and 450 nm, respectively. (**c**,**d**) In situ MB emission images for pure ZnO under the excitation wavelengths of 325 and 450 nm, respectively. Scan number: 200, scan time: 300 min.

We used a simple in situ PL emission characterization to further investigate the photo-degradation of MB with ZnO and 3%CuO/CeO_2_/ZnO under the excitation of specific wavelengths of UV (325 nm) and visible light (450 nm), as shown in Figure 6a–d. The strong peak located at ~650 nm is originated from frequency-doubled emission (Figure 6a,c). Figure 6a,b, respectively, display the MB in situ emission images for sample 3%CuO/CeO_2_/ZnO under the excitation of 325 and 450 nm, and the emission intensities of MB are found to significantly reduce with the increase of scan time. In sharp contrast, as displayed in Figure 6c,d, the MB emission intensity under 325 nm excitation for ZnO reduces slightly and, there is no obvious decrease observed under 450 nm excitation. The results intuitively suggest that the photocatalytic performance of sample 3%CuO/CeO_2_/ZnO was both enhanced in the UV and visible light region compared to pure ZnO. 

**Figure 7:**
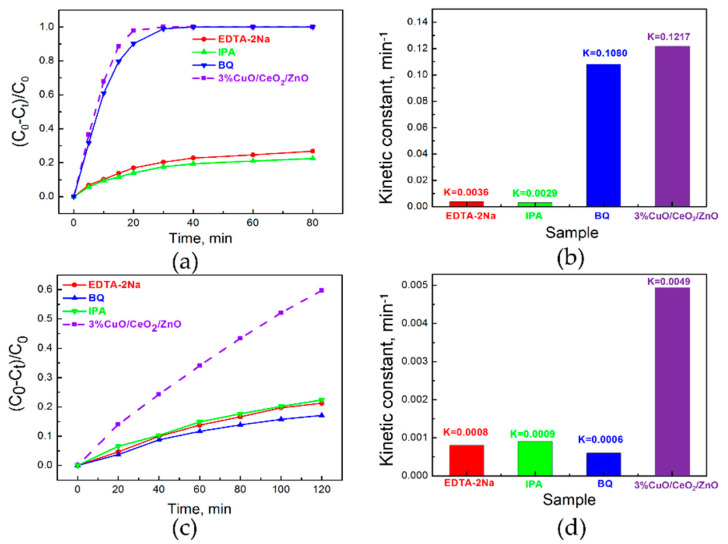
Degradation curves of MB with quenching agents and the corresponding kinetic constants under (**a**,**b**) UV light and (**c**,**d**) visible light.

To determine the effective active radicals in the photocatalytic reactions, quenching agents were added. As shown in Figure 7a,b, under UV light irradiation, the photocatalytic performance of sample 3%CuO/CeO_2_/ZnO (K = 0.1217 min^−1^) is dramatically deteriorated by EDTA-2Na (K = 0.0036 min^−1^) or IPA introduction (K = 0.0029 min^−1^), and the MB decomposition efficiency is slightly reduced by the addition of quenching agent BQ (K = 0.1080 min^−1^), so h^+^ and ·OH are considered to be the main active radicals, and ·O_2_^−^ plays a supplementary role in decomposing MB molecules under UV light. As can be seen from Figure 7c,d, when the solution with photocatalyst is irradiated under visible light (λ > 400 nm), the photocatalytic activity of sample 3% CuO/CeO_2_/ZnO (K = 0.0049 min^−1^) is reduced remarkably in the cases of EDTA-2Na (K = 0.0008 min^−1^), IPA (K = 0.0009 min^−1^), and BQ (K = 0.0006 min^−1^) addition, so h^+^, ·OH, and ·O_2_^−^ all play vital roles in promoting MB degradation under visible light irradiation. The results indicate that the enhanced visible light driven photocatalytic performance can be partly attributed to the photo-excited e^−^ of CuO that moves to the surface. 

Due to the low oxygen content in water, the promotion effect of O_2_^−^ on photo-degradation is presumed to be relatively weak. To adequately utilize the photo-excited e^−^, 1 mL hydrogen peroxide (H_2_O_2_, Chengdu Jinshan Chemical Reagent Co., LTD., Chengdu, China, ≥30%) was introduced to the system. The MB decomposition processes can be accelerated by the following reactions:

H_2_O_2_ + e^−^ → H_2_O +·OH + OH^−^ (6)

OH + MB → H_2_O + CO_2_↑ (7)

**Figure 8:**
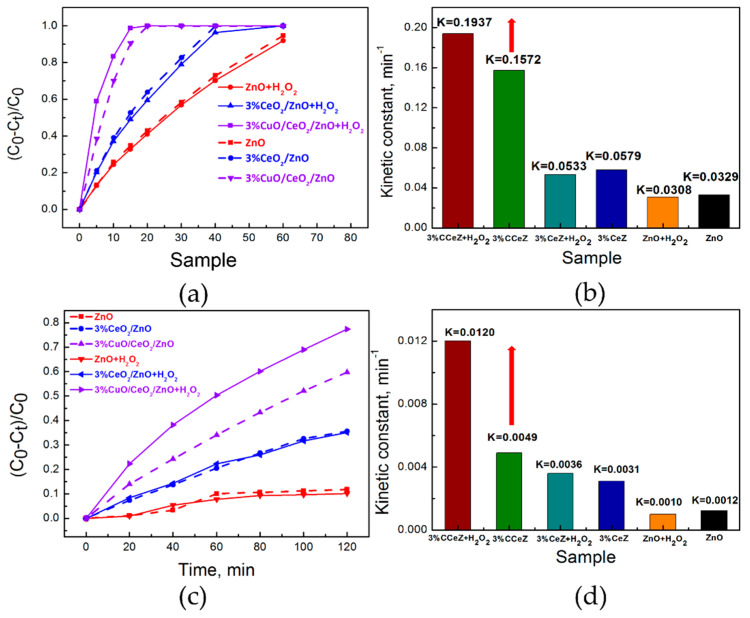
MB degradation curves and the corresponding kinetic constants for the as-prepared photocatalysts and photocatalysts-H_2_O_2_ under (**a**,**b**) simulated sunlight and (**c**,**d**) visible light.

As shown in Figure 8a–d, the MB decomposition efficiency for sample 3%CuO/CeO_2_/ZnO under simulated sunlight and visible light illumination is significantly enhanced by H_2_O_2_ introduction. The MB degradation K values for sample 3% CuO/CeO_2_/ZnO are increased from 0.1572 to 0.1937 min^−1^ under simulated sunlight and from 0.0049 to 0.0120 min^−1^ under visible light, respectively. H_2_O_2_, here, serves as an activating agent to make e^−^ be involved in reactions effectively, which further enhances the e^−^ utilization rate and therefore promotes the photocatalytic degradation. However, ZnO and sample CeO_2_/ZnO exhibit no obvious enhancement in photocatalytic efficiency with H_2_O_2_ introduction because the carriers distributed on these materials surface are mainly h^+^ under UV and visible light, as understood form SPV results. These experiments evidence the existence of photo-generated e^−^ of CuO on the surface of CuO/CeO_2_/ZnO heterostructures.

**Figure 9:**
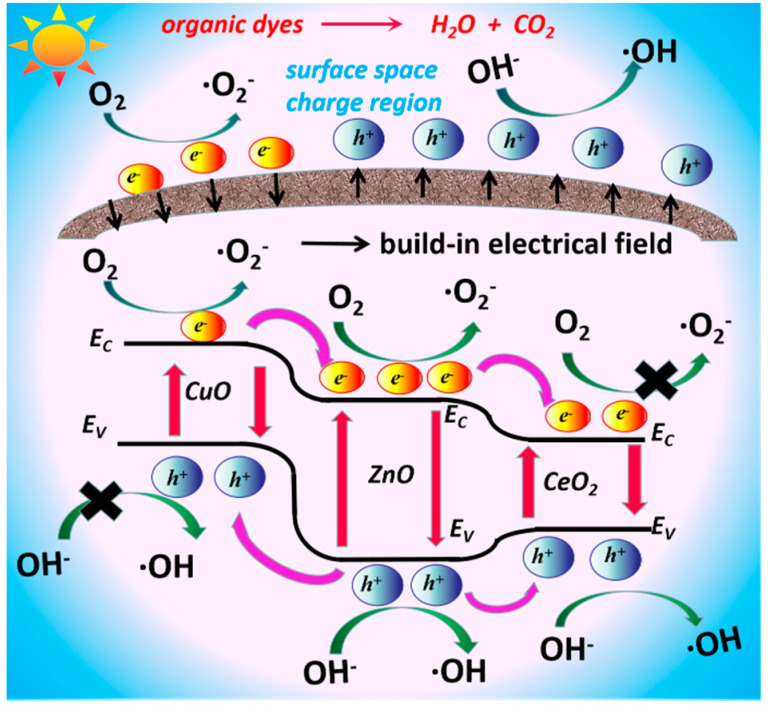
Graphical illustration of photo-degradation mechanism of CuO/CeO_2_/ZnO ternary heterostructure.

Since the VB edge potential of ZnO (+2.79 eV vs. standard hydrogen electrode, SHE) is more positive than CeO_2_ (+2.35 eV vs. SHE), and the CB edge potential of ZnO (−0.33 eV vs. SHE) is more negative than CeO_2_ (−0.23 eV vs. SHE) [16,26], the exciton transfer between ZnO and CeO_2_ is energetically favourable, which is responsible for the significantly reduced PL emission of ZnO after the introduction of CeO_2_. The oxidation potential of holes in both VBs of ZnO and CeO_2_ are large enough to oxidize OH^-^ to ·OH (+1.99 V vs. SHE). However, comparing the CBs of ZnO and CeO_2_, only the reduction potential of photo-generated electrons in the CB of ZnO can reduce O_2_ to·O_2_^−^ (−0.33 V vs. SHE) [16,26]. As for the CeO_2_/ZnO heterostructure, the oxidization reactions participated by the photo-generated h^+^ of CeO_2_ and the enhanced visible light response, as revealed from the SPV results, are the most probable reasons for the enhancement of the photo-activity of ZnO. However, from the photocatalytic results, the photocatalytic efficiency of CeO_2_/ZnO was only ~1.8, ~1.8 and ~2.6 times higher than ZnO under simulated sunlight, UV light and visible light, respectively. Theoretically, the excitons transmitted from ZnO to CeO_2_ may result in high recombination rate of electrons and holes in CeO_2_, so CeO_2_ in the system, which is unable to participate in the photocatalytic reaction efficiently. When CuO is introduced into the system, the visible light absorption is significantly enhanced, the recombination of e^−^/h^+^ pairs is further inhibited due to the favourable charge transfer between CuO and ZnO [16,29], which leads to a much stronger UV light response in SPV, and the negative signal in the visible light region originated from CuO arises. As CuO is a p-type semiconductor with a downward surface band bending, the direction of the build-in electrical field in the surface space charge region is from surface to bulk, so photo-excited e^−^ will move to the surface of the catalyst [27]. Opportunely, though the VB edge potential of CuO (+0.67 V vs. SHE) is more negative than the standard redox potential E = (OH^−^/OH), which causes the photo-generated h^+^ of CuO cannot oxidize OH^-^ to OH, the CB edge potential of CuO is −0.73 eV so that the photo-excited e^−^ in the CB of CuO can react with O_2_ to form ·O_2_^−^ [16,30]. So, when CuO/CeO_2_/ZnO is under visible light illumination, the electrons in CuO are excited from VB to CB and migrate to the catalyst surface and then are involved in photocatalytic reactions, which can be evidenced from photocatalytic experiments. These characteristics are the main key factors for the further enhancement of photocatalytic performance under UV and visible light irradiation. That is to say, CuO makes up for the deficiency of CeO_2_/ZnO to a great extent. Especially, the sample 3% CuO/CeO_2_/ZnO possesses the optimal photocatalytic properties and exhibits a high performance in various organics degradation because of the strongest visible light absorption, the highest photo-responses to UV and visible light, and the most efficient photo-excited e^−^/h^+^ pairs separation efficiency. Based on the above experiments and discussion, the most likely photocatalytic mechanism of the designed CuO/CeO_2_/ZnO nano-structure with complementary band structures is depicted in Figure 9.

## 4. Conclusions

To conclude, we fabricated a ternary CuO/CeO_2_/ZnO photocatalyst by a facile two-step sol-gel method. By introducing CuO and CeO_2_ as effective visible light harvesters and responses, and facilitating photo-generated charge carrier separation within the rationally designed heterostructure, we can progressively enhance the photocatalytic performance of ZnO under UV and visible light excitation. The photocatalytic efficiency of ZnO can be stepwise boosted by ~1.8 and ~4.8 times under simulated sunlight, and ~2.6 and ~4.1 times under visible light illumination with CeO_2_ coupling and CuO, CeO_2_ co-coupling, respectively. Dissimilarly, CeO_2_ mainly plays a role in performing photocatalytic oxidation by taking advantage of valence band h^+^ under UV and visible light excitation, while CuO introduction facilitates the photo-excited charge separation effectively, furnishes much higher visible light absorption and promotes photocatalytic process by participating in reactions with photo-generated e^−^ that moves to the surface. This work offers an easy, low-cost strategy to construct a photocatalyst containing multiple components with a clear division of work.

## Figures and Tables

**Table 1:** Main reagent masses used in synthetic processes of CuO/CeO_2_/ZnO.

Samples	Zn(CH_3_COO)_2_·2H_2_O/g	Ce(NO_3_)_3_·6H_2_O/g	Cu(CH_3_COO)_2_·H_2_O/g
ZnO	4.4343	0.0000	0.0000
3%CeO_2_/ZnO	4.4343	0.2667	0.0000
1%CuO/CeO_2_/ZnO	4.4343	0.2667	0.0403
3%CuO/CeO_2_/ZnO	4.4343	0.2667	0.1210

**Table 2:** Microstructural parameters and crystallite size of as-synthesized samples (θ, d, a, c, and D represent the diffraction angle, interplanar spacing, lattice parameters a and c, and the diameters of ZnO, respectively).

Samples	2θ (100)/°	d(100)/nm	d(002)/nm	d(101)/nm	a/nm	c/nm	D/nm	Cell Vol/Å^3^
ZnO	31.84	0.2808	0.2598	0.2473	0.3243	0.5448	36.4	49.6
3%CeO_2_/ZnO	31.87	0.2805	0.2595	0.2471	0.3239	0.5439	32.7	49.4
1%CuO/CeO_2_/ZnO	31.92	0.2797	0.2590	0.2465	0.3230	0.5429	30.3	49.0
3%CuO/CeO_2_/ZnO	31.95	0.2795	0.2585	0.2459	0.3227	0.5416	28.6	48.8
